# Association of cytochrome P450 2C19 polymorphisms with coronary heart disease risk: A protocol for systematic review and meta analysis

**DOI:** 10.1097/MD.0000000000023652

**Published:** 2020-12-11

**Authors:** Yongxin Yang, Yaping Zhang, Ming Ren, Yonglan Wang, Zhuoma Cairang, Rongxiang Lin, Haixia Sun, Jianju Liu

**Affiliations:** a Department of Cardiology, The People's Hospital of Qinghai Province; b Department of Cardiology, Qinghai University Affiliated Hospital; c Echocardiography Room, The People's Hospital of Qinghai Province, Xining, China.

**Keywords:** coronary heart disease, meta-analysis, polymorphism, polymorphisms in the cytochrome P450 2C19

## Abstract

**Background:**

Polymorphisms in the cytochrome P450 2C19 (CYP2C19) gene have been reported to be associated with coronary heart disease (CHD), but the results were not consistently analyzed among different patient groups. To derive a more precise estimation of these associations, we will conduct a meta-analysis to investigate the polymorphisms of CYP2C19 in all published studies.

**Methods:**

Electronic databases (Google Scholar, ISI Web of Science, Pubmed, Embase, China National Knowledge Infrastructure, Wanfang, and China Biological Medicine) will be used to search clinical case-control or cohort studies about CYP2C19 polymorphism and CHD published until November 2020. Two reviewers will independently select the study, extract the data, and evaluate the quality of the study. Odds ratios with 95% confidence interval will be used to evaluate the strength of the association between the CYP2C19 polymorphism and CHD susceptibility under 4 genetic models. Subgroup analysis will be conducted by different ethnicity and genotyping method. Sensitivity analysis will be performed via sequentially omitting each of the included studies 1 at a time. Begg funnel plots and Egger test will be used to examine the potential publication bias. All the statistical analyses will be performed using Review Manager 5.3 and Stata 12.0.

**Results:**

This study will provide a better understanding of the association between CYP2C19 polymorphisms and coronary heart disease risk.

**Conclusion:**

The publication of this protocol will minimize the possibility of bias due to post hoc changes to the analysis protocol, thus helping to obtain reliable evidence.

**OSF registration number:**

DOI 10.17605/OSF.IO/R7U93

## 1 Introduction

Coronary heart disease (CHD) is one of the most common cardiovascular diseases in the world, which is characterized by arteriosclerosis and coronary artery stenosis and even obstruction. The incidence and mortality of CHD have been showing a downward trend since the 1970s, mainly due to the improvement of prevention and treatment measures and the reduction of major risk factors.^[1,2]^ However, the disease is still being the main cause of death and disability among people aged 35 and older, and seriously endangers human health.^[3–5]^ In particular, individuals with acute myocardial infarction have a higher risk of recurring CHD events, recurring myocardial infarction, and death.^[6]^ It is estimated that more than 9.4 million people worldwide died of ischaemic heart disease in 2016.^[7]^ In China, the mortality rate of CHD in urban residents was 115.32/100,000, and that in rural residents was 122.04/100,000.^[8]^ Whether in developed or developing countries, the management of CHD every year is related to huge long-term medical expenses.^[9]^

The complex pathophysiology of CHD depends on a large number of lifestyle and environmental risk factors of its individual. It has been investigated through some studies that provide key information on primary and secondary preventive measures.^[10]^ It is well known that the traditional risk factors for CHD include age, smoking, elevated blood pressure, dyslipidemia, diabetes mellitus, irregular eating, obesity, and insufficient physical activity.^[11]^ However, in clinical practice, many patients have been diagnosed with CHD, but there is no clear explanation for their occurrence and progress in the case of premature coronary artery involvement to life-threatening conditions caused by traditional risk factors. Familial aggregation of CHD has been investigated for a long time. Twin studies showed that the heritability of CHD is estimated to be between 41% and 77%.^[12]^ More recently, researchers have proposed genetic risk factors for CHD, which can be identified by the robust genome-wide association studies.^[13]^ In addition, genome-wide association studies showed that there are numerous candidate genes and considerable single nucleotide polymorphisms remarkably associated with CHD, such as VEGF,^[14]^ SCARB1,^[15]^ ADAMTS-7,^[16]^ SCARB1,^[17]^ VEGFA,^[18]^ MADD-FOLH1,^[19]^ CYPs,^[20]^ and APOC3.^[21]^

Cytochrome P450 2C19 (CYP2C19), as one of the main cytochromes P450s drug metabolic enzymes in human body, is encoded by chromosome 10 and expressed in the human liver.^[22]^ It is the most polymorphic member of CYP2 C subfamily.^[23]^*CYP2C19* gene mutation can change the activity of human related enzymes, affect the metabolic process of related drugs, and lead to cardiovascular events in patients with CHD. For example, the Allelic variation classified the population according to the catalytic activity of CYP2C19 to ultra-rapid, extensive and poor phenotypes for drug clearance.^[24]^ CYP2C19 gene polymorphism has been proved to be a compelling predictor of clopidogrel resistance, which can lead to failure in the treatment of CHD.^[25]^ Patients with loss-of-function allelic variants (CYP2C19 ∗ 2 and CYP2C19 ∗ 3) are more prone to thromboembolic events.^[26]^ In Zhang's study, the adverse impact of CYP2C19∗2 polymorphisms was found not only in the risk of CHD, but also in the adverse clinical outcomes in CHD patients during a long follow-up period.^[27]^ Ercan reported that among Turkish people, smokers with CYP2C19∗2 mutations have a 3.7 times risk of CHD.^[28]^ The CYP2C19 ∗ 3 genotype was found to be significantly more common in Chinese Uighur populations with CHD.^[29]^

On the basis of this information, these studies have suggested that the single-nucleotide polymorphism in the *CYP2C19* gene may be involved in the occurrence and development of CHD. Unfortunately, the results of these studies were not consistently analyzed among different patient groups. Therefore, it is necessary to conduct a meta-analysis of all eligible studies to obtain more convincing evidence on the association of CYP2C19 polymorphism and CHD susceptibility.

## 2 Methods/design

### 2.1 Study registration

The retrospective review does not involve the evaluation of patients’ personal information or rights, so ethical approval is not required. It cannot be ignored that we have registered the protocol in the Open Science Framework (OSF) in advance, and the registration number is DOI 10.17605/OSF.IO/R7U93. In addition, this systematic review and meta-analysis will be reported in accordance with the preferred reporting items for systematic reviews and meta-analysis protocols 2015.^[30]^

### 2.2 Inclusion criteria

The inclusion criteria will be made based on the PICOS principle (participants, intervention, control, outcome, and study type):

(1) Types of studies: all case-control studies or cohort studies that evaluated the association between CYP2C19 polymorphisms and CHD risk will be incorporated in our review. No restriction will be put on the publication date of the studies.
(2) Types of participants: the present meta-analysis will include subjects with CHD, and the diagnosis should be made in accordance with well-established guidelines. The control subjects should be defined as healthy individuals. Regardless of age, gender, or country.
(3) Studies that presented original data, or provided the genotypic frequency of both case and control samples or had odds ratios (ORs) with 95% confidence interval (CI) values.
(4) Primary outcome: the association between CYP2C19 polymorphisms and risk of CHD.

### 2.3 Exclusion criteria

Studies will be excluded from the meta-analysis according to the following criteria:

(1) review articles,
(2) in vitro or animal study,
(3) case reports,
(4) conference abstracts,
(5) Newcastle–Ottawa scale (NOS) score is less than 6,
(6) studies that did not provide allele frequencies or genotypic for samples,
(7) studies on the relationship between *CYP2C19* gene polymorphism and non-coronary heart disease,
(8) overlapping or duplicate studies.

### 2.4 Search strategy

Several online databases including Pubmed, ISI Web of Science, Embase, Google Scholar, Wanfang, China Biological Medicine, and China National Knowledge Infrastructure will be searched up to November, 2020. Medical subject headings (MeSH) and synonymous free texts will be combined to improve the comprehensiveness and sensitivity of literature retrieval. The language will be restricted to Chinese and English. The following keywords will be used: (“coronary heart disease” OR “CHD” OR “coronary artery disease” OR “CAD”) and (“Cytochrome P-450 CYP2C19” OR “CYP2C19”) and (“polymorphism” OR “variant” OR “genotype” OR “mutation”). The search strategy for PubMed is shown in Table 1, and the corresponding keywords will be used in the Chinese databases. In addition, we will supplement this search by manually scanning the cross-references of related articles.

**Table 1:** Search strategy used in PubMed database.

Number	Search items
#1	Search: “Cytochrome P-450 CYP2C19”[Mesh]
#2	Search: ((((((((Cytochrome P-450 CYP2C19[Title/Abstract]) OR (CYP2C19[Title/Abstract])) OR (CYP2C19, Cytochrome P-450[Title/Abstract])) OR (Cytochrome P 450 CYP2C19[Title/Abstract])) OR (P-450 CYP2C19, Cytochrome[Title/Abstract])) OR (CYPIIC19[Title/Abstract])) OR (S-Mephenytoin 4’-Hydroxylase[Title/Abstract])) OR (S Mephenytoin 4’ Hydroxylase[Title/Abstract])) OR (CYPIIC19[Title/Abstract])
#3	Search: #1 OR #2
#4	Search: “Polymorphism, Genetic”[Mesh]
#5	Search: ((((((((((Polymorphism, Genetic[Title/Abstract]) OR (Polymorphism[Title/Abstract])) OR (Genetic Polymorphisms[Title/Abstract])) OR (Genetic Polymorphism[Title/Abstract])) OR (Polymorphisms[Title/Abstract])) OR (single nucleotide polymorphism[Title/Abstract]))) OR (SNP[Title/Abstract])) OR (variant[Title/Abstract])) OR (variation[Title/Abstract])) OR (mutation[Title/Abstract])
#6	Search: #4 OR #5
#7	Search: “Coronary Disease”[Mesh]
#8	Search: ((((((Coronary Disease[Title/Abstract]) OR (Disease, Coronary[Title/Abstract])) OR (Coronary Heart Disease[Title/Abstract])) OR (Coronary Heart Diseases[Title/Abstract])) OR (CHD[Title/Abstract])) OR (coronary artery disease[Title/Abstract])) OR (CAD[Title/Abstract])
#9	Search: #7 OR #8
#10	Search: #3 AND #6 AND #9

### 2.5 Data collection and analysis

#### 2.5.1 Selection of studies

**Figure 1:**
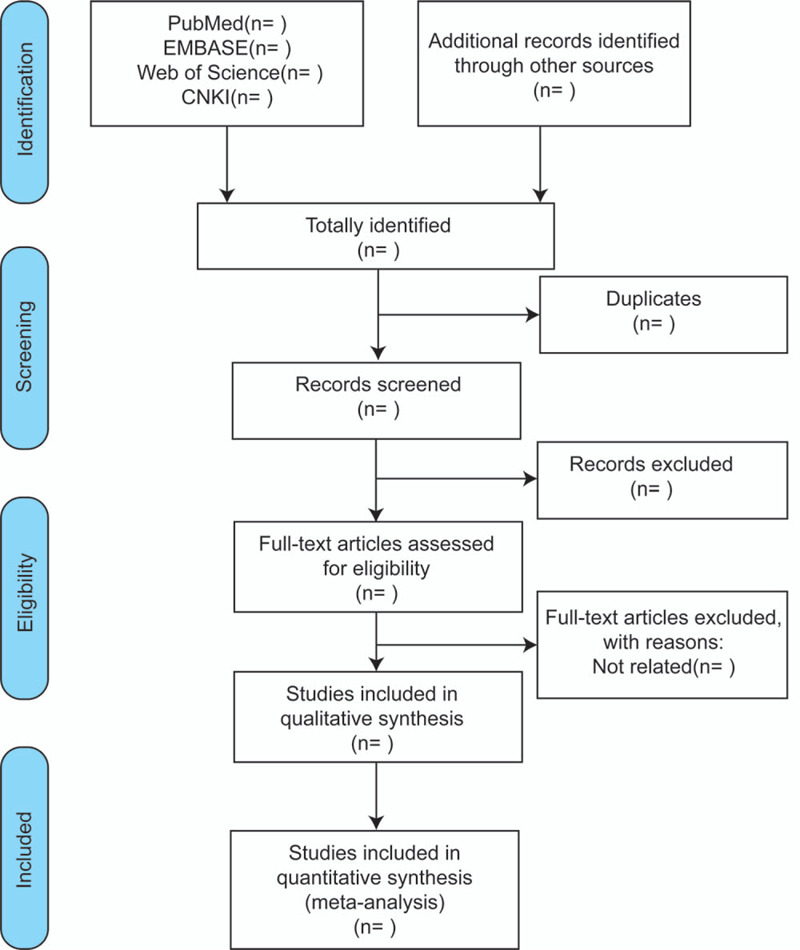
Flow chart of study selection.

The researchers of our team have received professional training on the purpose and process of the review in advance. Two reviewers (Yongxin Yang and Yaping Zhang) will perform the selection process independently, with cases of disagreement will be resolved by adjudication by a third reviewer (Ming Ren). The screening process of the article includes reading the title first, then the abstract and the full text to determine whether it meets the inclusion criteria. The researchers will record the reasons for excluding each study in light of the preferred reporting items for systematic reviews and meta-analysis guidelines and report the screening results.^[31]^ The flowchart is shown in Figure 1.

#### 2.5.2 Data extraction

Each publication will be evaluated thoroughly, and data will be extracted independently by 3 researchers, including year of publication, surname of the first author, ethnicity of each study population, country, mean age, gender, numbers of subjects, genotyping methods, allele frequencies and genotype distribution of CYP2C19 polymorphisms. Group discussions will be conducted to resolve any disagreements in the extraction process. If the data for a paper is incomplete or unconvincing, we will try to contact the author via email. In addition, the Hardy–Weinberg equilibrium (HWE) of genotype distributions in the control group will also be examined.

#### 2.5.3 Study quality assessment

Two reviewers (Yonglan Wang and Zhuoma Cairang) will independently assess the quality of all included studies based on NOS, which is specifically used to assess the quality of observational studies.^[32]^ When they encounter disagreement, they will resolve these issues with the help of a third member (Rongxiang Lin). As we all know, the NOS values arrange from 0 to 9. The quality of publication below 6 stars is low, and the quality of research above 6 stars is high. Only studies with more than 6 stars will be included in our study.

#### 2.5.4 Statistical analysis

The ORs with their corresponding 95% CIs will be used to assess the strength of the association between CYP2C19 polymorphisms and CHD susceptibility. The pooled ORs will be conducted for 4 genetic (allelic genetic model: T versus C; recessive genetic model: TT versus CT + CC; dominant genetic model: TT + CT versus CC; and additive model: TT versus CC. “T” and “C” represent the mutant allele and the wild-type allele, respectively). Then the most reasonable genetic model of inherence will be identified based on the relationships between the 4 pairwise comparisons. After identifying the potential genetic model, the genotype count will be collapsed into 2 categories to obtain a combined result. The significance of the pooled ORs will be determined by *Z*-test, with *P* < .05 considered statistically significant. In addition, the Fisher exact test will be used to assess the deviation of CYP2C19 polymorphism frequency from the expected values under the HWE among healthy controls. When *P* < .05, the study is considered to be inconsistent with HWE. *χ*^2^ test-based *Q* statistic and *I*^2^ will be applied to assess the overall heterogeneities. If *I*^2^ values < 50% and *P* > .05, heterogeneity is deemed to be low, and a fixed-effect model will be selected for data integration. Otherwise, a random-effect model will be used.^[33,34]^ In our study, all statistical analyses will be conducted by using Stata version 12.0 (Stata Corporation, College Station, TX) and Review Manager 5.3 (Cochrane Collaboration, Oxford, UK).

#### 2.5.5 Assessment of heterogeneity

If there is obvious heterogeneity among studies, we will conduct the meta-regression analysis, subgroup analysis or sensitivity analysis to explore the potential sources of heterogeneity. The confounding factors include ethnicity, year of publication, type of study, total sample size, and RR (ratio of case size to control size).

#### 2.5.6 Subgroup analysis

Subgroup analysis will be conducted by different ethnicity, genotyping method, total sample size and deviation from HWE, and so on.

#### 2.5.7 Sensitivity analysis

The sensitivity analysis will be carried out by sequentially omitting each of the included studies 1 at a time to measure the reliability and robustness of the research results.

#### 2.5.8 Assessment of publication biases

Funnel plots will be used to evaluate publication bias if more than 10 eligible studies are included, and the criterion was whether the funnel plot is symmetric or not. If the funnel chart is asymmetric, there may be publication bias. What's more, Begg rank correlation test and Egger test will also be used for checking the potential publication bias, and a *P*-value smaller than .05 is considered statistically significant.

#### 2.5.9 Grading the evidence quality

GRADE method will be used to assess the quality of evidence for our findings.^[35]^ The quality of evidence can be divided into 4 levels: high, medium, low, and very low quality. Researchers should consider certain factors that may reduce the quality of the evidence, such as the heterogeneity between groups, estimate precision of effect, publication bias, and evidence directness. Moreover, large magnitude effect and dose-response gradient that increase the quality of evidence should also be given enough attention.

## 3 Discussion

Cardiovascular disease is the largest cause of death in the world, especially CHD manifested by angina pectoris or myocardial infarction. Although the mortality rate of CHD has declined, it still causes one-third of all deaths over the age of 35 in the past few decades.^[36]^ The disease is regarded as a complex multifactorial disease caused by the interaction between multiple environmental and genetic factors.^[37]^ Traditional risk factors are considered to be the cause of most CHDs, although 15% to 20% of patients have no identified risk factors, making it impossible to prevent adverse cardiovascular events with adequate treatment. Early identification and intervention strategies are essential to reduce the incidence and mortality of CHD in high-risk population. Identifying the genetic components of the disease is the important area in cardiovascular disease research, because clarifying the genes related to CHD will affect all efforts towards understanding of the mechanism level, its prevention and treatment. Fortunately, genome-wide association studies have confirmed some genes related to the susceptibility to CHD in different populations around the world, of which the *CYP2C19* gene is well known.

So far, although many researchers have focused on the relationship between *CYP2C19* gene polymorphism and CHD susceptibility, there has been no systematic assessment of the cumulative evidence for this association. In this study, we will conduct a systematic review and meta-analysis to combine results from numerous studies and generate a more reliable estimate of risk association to provide guidance for the prevention and treatment of coronary heart disease. The strengths of this study include the following aspects:

(1) large datasets from all recent eligible studies will be incorporated;
(2) for the exploration of heterogeneity, we will try to avoid post-group subgroup analysis; and
(3) sensitivity analysis of each genetic model will be performed to improve the reliability of the results.

Therefore, publishing the protocol will avoid the potential bias related to data mining as much as possible and will help to obtain convincing evidence.

## Author contributions

**Conceptualization:** Yongxin Yang, Yaping Zhang.

**Investigation:** Ming Ren, Yonglan Wang.

**Supervision:** Yongxin Yang.

**Writing – original draft:** Zhuoma Cairang, Rongxiang Lin.

**Writing – review & editing:** Haixia Sun, Jianju Liu.
